# Associations between social support and help-seeking intention regarding music performance anxiety among Chinese music students: mediation via resilience and self-compassion

**DOI:** 10.3389/fpsyg.2026.1787708

**Published:** 2026-02-12

**Authors:** Jian Sun, Lijuan Zhong, Xuan Zhou, Zhan Li, Qian Liu, Yanchang Liu

**Affiliations:** 1 School of Music and Dance, Xihua University, Chengdu, China; 2 College of Arts, Guangxi Minzu University, Nanning, China

**Keywords:** help-seeking intention, music performance anxiety, resilience, self-compassion, social support

## Abstract

**Introduction:**

Music performance anxiety (MPA) is a common challenge in higher music education, often undermining students’ confidence, performance quality, and mental health. However, research on help-seeking related to MPA among music students remains limited. Social support is known to buffer stress and promote help-seeking, while self-compassion and resilience may serve as key psychological mechanisms linking social support to help-seeking intentions. This study examined how social support relates to both formal and informal help-seeking intentions regarding MPA among Chinese music students, focusing on the mediating roles of self-compassion and resilience.

**Methods:**

A cross-sectional online survey was conducted in March 2025 among 684 students from Sichuan, China. Validated scales measured social support, self-compassion, resilience, and help-seeking intentions. Mediation analyses were performed using structural equation modeling.

**Results:**

The present study found that social support was not directly related to formal help-seeking intention, but its indirect effects through resilience and self-compassion were significant, indicating full mediation. For informal help-seeking, social support had both direct and indirect effects via resilience and self-compassion, suggesting partial mediation.

**Discussion:**

The findings indicate that social support facilitates help-seeking through both self-compassion and resilience. Strengthening peer and teacher support systems, incorporating self-compassion and resilience training into performance pedagogy, may enhance students’ readiness to seek help. Future longitudinal research should verify these pathways and examine actual help-seeking behaviors.

## 1 Introduction

In higher music education, music performance anxiety (MPA) is a common phenomenon (Nielsen et al., 2017). For university music students, the need to improve their technical skills and take part in frequent performances, examinations, and assessments often brings constant pressure. Studies have shown that MPA is not just a feeling of nervousness. It involves emotional, cognitive, physical, and behavioral reactions, such as a fast heartbeat, muscle tension, lack of concentration, and negative self-evaluation (Papageorgi, 2020; Kenny, 2023). For many music students, MPA can reduce the quality of their performance and weaken confidence (Wang and Yang, 2024). Over time, it may also affect their mental health, professional goals, and motivation to continue studying music (Du et al., 2025). As they are both learners and performers, many students have not yet learned effective ways to manage MPA in performance situations. These students need proper support and help to cope with MPA and adjust to university life. If they do not receive help, MPA may become worse in important performances and may even develop into a long-term psychological problem (Kinney et al., 2025).

Seeking help is a protective factor against MPA (Rickwood et al., 2005). However, this behavior is not common among music students. Many students prefer to handle MPA on their own because they fear being seen as weak or doubt the usefulness of professional services (Gulliver et al., 2010). Limited awareness of available resources and low confidence in seeking help further decrease their willingness to reach out for support (Shim et al., 2022). Help-seeking intention plays a crucial role in understanding students’ responses to MPA because it represents the motivation and readiness to seek help before any actual behavior occurs (Baldofski et al., 2024). A strong intention to seek help often predicts future help-seeking behavior, making it a key indicator for early intervention (Adams et al., 2022). In this study, formal help-seeking intention (FHSI) refers to students’ willingness to ask for help from psychologists, counselors, mental health professionals, teachers, or other university services, while informal help-seeking intention (IHSI) refers to seeking support from friends, peers, or family members (Lu et al., 2025b). Examining help-seeking intention rather than behavior provides insight into potential barriers and facilitators before students take action, which is essential for designing preventive and supportive strategies (Zhao et al., 2025). Understanding these factors is important for developing strategies that encourage music students to seek appropriate support and better manage MPA.

According to the Stress and Coping Theory (SCT; Lazarus and Folkman, 1984), people use different coping resources to deal with stress, and these resources can shape how they respond to stressful events. In the case of MPA, seeking help from others can be viewed as a positive and active coping response (Kinney et al., 2025). Social support, as an important external coping resource, can provide emotional comfort, practical advice, and a sense of belonging (Taylor, 2012; Acoba, 2024). For music students, such support often comes from parents, teachers, and peers who understand the challenges of performance and study. When students feel supported by people around them, they are more likely to view help-seeking as acceptable and useful (Vidourek et al., 2014; Li et al., 2025). Previous research has shown that social support increases students’ confidence to reach out for both formal and informal help when needed. Empirical studies have consistently confirmed this association. For instance, social support from parents, friends, and peers has been found to play a key role in promoting help-seeking behaviors among young people (Wahlin and Deane, 2012; Maiuolo et al., 2019). A systematic review also identified social support as one of the strongest predictors of help-seeking (Gulliver et al., 2010). Moreover, research has shown that interpersonal support can reduce MPA and promote help-seeking among music students (Zhang and Jenatabadi, 2024). Therefore, in the context of MPA, social support can act as a protective factor that encourages adaptive coping and strengthens students’ willingness to seek help.

Within SCT framework, self-compassion can be viewed as an internal psychological resource that complements external support. It refers to an individual’s capacity for self-empathy, openness to emotional distress, and the ability to bestow care and kindness upon oneself (Neff, 2003). For music students, who are often under pressure to perform perfectly, self-compassion can help them respond to MPA and mistakes with understanding rather than shame (Zhang and Jenatabadi, 2024). Social support may foster self-compassion by creating a sense of emotional safety (Ross and Ross, 2025). When students experience acceptance and empathy from others, they are more likely to treat themselves with the same understanding (Ross and Ross, 2025). In turn, higher self-compassion can reduce self-blame and increase openness to seeking help when facing MPA. Empirical research has consistently shown that individuals with higher levels of self-compassion demonstrate better emotional adjustment, stronger self-regulation, and greater engagement in adaptive coping behaviors such as help-seeking (Biber and Ellis, 2019; Chiang et al., 2021). Recent research grounded in Stress and Coping Theory further suggests that social support and self-compassion jointly promote adaptive outcomes among university students by facilitating stress management and psychological adjustment (Wang and Wang, 2025). Moreover, self-compassion has been linked to a higher likelihood of seeking professional help when experiencing stressful events (Mak et al., 2011; Brenner et al., 2018). Therefore, self-compassion may be a mediating factor that explains how social support influences students’ willingness to seek help for MPA. It helps translate supportive experiences into greater openness and confidence in coping with MPA.

From the perspective of SCT (Lazarus and Folkman, 1984), resilience is viewed as an inner strength that helps individuals adjust to difficulties, recover after failure, and keep emotional balance under stress (Lu et al., 2024a). It encourages people to face problems with positive coping approaches, enabling them not only to endure challenges but also to learn and grow from them (Chen et al., 2023; Scheffert and Henson, 2025). Studies have shown that individuals with higher resilience tend to adopt more effective coping methods and are more willing to seek help when dealing with mental health concerns or performance-related pressure (Macía et al., 2021). Social support can enhance resilience by offering encouragement, stability, and a feeling of connection (Hanımoğlu, 2025). A supportive environment builds confidence in one’s ability to handle stress and helps people see help-seeking as a proactive and constructive step rather than a sign of weakness (Wiedermann et al., 2023). Research has consistently found that social support is positively linked with resilience among both adolescents and individuals exposed to stress (Sippel et al., 2015). More recent findings also suggest that resilience can act as a bridge between perceived social support and the intention to seek professional help. For instance, a study involving young Australians found that resilience mediated the relationship between social support and help-seeking intention (Ishikawa et al., 2023). In the context of MPA, students who feel supported by teachers, peers, or family members are likely to build stronger resilience. This inner capacity allows them to manage anxiety more effectively and increases their openness to seek professional or peer help when necessary.

Grounded in SCT (Lazarus and Folkman, 1984), the present study conceptualizes help-seeking intention as an adaptive coping outcome shaped by cognitive appraisal processes. In the context of MPA, students first engage in primary appraisal by perceiving performance demands as threatening or stressful. During secondary appraisal, individuals evaluate the controllability of MPA and the availability of coping resources (Li et al., 2025). At this stage, social support functions as a key external coping resource, enhancing perceived controllability and available coping assistance (Ross and Ross, 2025). Importantly, within the Chinese cultural context, strong face concerns and stigma surrounding psychological help may weaken the direct translation of social support into formal help-seeking. Under such conditions, the effects of social support may be more likely to operate indirectly, through the activation of internal psychological resources, providing a theoretical basis for examining self-compassion and resilience as mediating mechanisms.

Anchored in SCT and the current literature review, the present study aims to investigate the association between social support and FHSI/IHSI regarding MPA among Chinese music students. It also tests the mediating role of self-compassion and resilience between such association. The following hypotheses have been formulated: (H1) self-compassion would significantly mediate the association between social support and IHSI regarding MPA; (H2) resilience would significantly mediate the association between social support and IHSI regarding MPA; (H3) self-compassion would significantly mediate the association between social support and FHSI regarding MPA; (H4) resilience would significantly mediate the association between social support and FHSI regarding MPA.

## 2 Methods

### 2.1 Participants and data collection

A cross-sectional survey was conducted between March 2025 among students from four public music conservatories in Sichuan Province, China. These institutions are undergraduate-level higher education institutions offering accredited music programs and play a similar role in regional music education. Student recruitment is primarily regional, with some national enrollment, and all four conservatories offer comparable professional tracks, including music performance, music education, composition, and related music disciplines. No systematic differences in institutional level or student professional composition were identified across the four institutions.

Data were collected anonymously through Wenjuanxing (www.wjx.cn), an online survey platform widely used for academic research in China. The purpose, background, and confidentiality of the study were clearly stated on the cover page of the online questionnaire. Participants were informed that their participation was voluntary, and they could withdraw from the study at any time without any negative consequences. Informed consent was obtained electronically before the participants began the survey. This study was approved by the Research Ethics Committee of Xihua University (Approval No. XH250124-01).

Participants were recruited through official student communication channels of the participating conservatories, such as class-based online groups and departmental student networks. Eligibility criteria required participants to be currently enrolled undergraduate or postgraduate students majoring in music-related disciplines. At the beginning of the questionnaire, participants were asked to confirm their enrollment status, institution, and major. Responses that did not meet these inclusion criteria were excluded from the analysis.

A convenience sampling strategy was employed across the four conservatories to enhance sample heterogeneity and ecological validity within the regional context. A total of 728 music students initially participated in the survey. Data from 44 participants were excluded due to unrealistically short completion times or logical inconsistencies among item responses. After data cleaning, 684 valid questionnaires were retained for analysis, representing the final sample of the study.

### 2.2 Measures

#### 2.2.1 Background factors

Background factors were collected including age, sex, undergraduate levels, perceived family financial situation, and single-parent family.

#### 2.2.2 Social support

Social support was measured by using three-item Social Support Scale assessing perceived emotional support, instrumental support, and affirmation from families, friends, and peers (Yu et al., 2022). This brief measure has demonstrated satisfactory reliability and validity among Chinese samples (Yu et al., 2022). An example item is “When you need to talk with someone or emotional support, your families, friends, and peers would give you adequate support”. Participants rated each item on a seven-point Likert scale (0 = strongly disagree to 7 = strongly agree), with higher scores indicating greater perceived social support. The Cronbach’s *α* was 0.85 in this study.

#### 2.2.3 Resilience

Resilience was assessed by using the 2-item abbreviated version of the Connor-Davidson Resilience Scale (Vaishnavi et al., 2007). The Chinese adaptation has shown satisfactory psychometric properties among adult samples (Ni et al., 2016; Zhang et al., 2021). The two items were “able to adapt to change” and “tend to bounce back after illness or hardship” (0 = not true at all to 4 = true nearly all the time). The Cronbach’s α was 0.83 in this study.

#### 2.2.4 Self-compassion

Self-compassion was assessed by using the Self-compassion Scale Short Form (Raes et al., 2011). The Chinese version showed acceptable properties and has been validated and used among students and medical workers in previous Chinese studies (Meng et al., 2019). A sample item was “When I go through tough times, I give myself the care and love I need”. Each item is rated on a five-point Likert scale (1 = almost never to 5 = almost always). Higher scores indicated higher levels of self-compassion. The Cronbach’s alpha was 0.83 in this study.

#### 2.2.5 Informal help-seeking intention (IHSI)

Informal Help-seeking Intention Scale was used to assess IHSI (Lu et al., 2025b). It consists of two items, including “If you were experiencing music performance anxiety, how likely would you seek help from parents?” and “If you were experiencing music performance anxiety, how likely would you seek help from peers and/or friends?”. Each item is rated on a five-point Likert scale (0 = very low to 5 = very high). The Cronbach’s alpha was 0.84 in this study.

#### 2.2.6 Formal help-seeking intention (FHSI)

FHSI was assessed using a single-item measure (Lu et al., 2024b). The item was “If you were experiencing music performance anxiety, how likely would you seek help from psychologists, counselors, mental health professionals, teachers, or other university services?” The item was rated on a five-point Likert scale (0 = very low to 5 = very high).

### 2.3 Data analysis

The Harman single-factor was employed to assess common method bias. It indicates that the common method bias is not a concern if the strongest factor explains less than 40% of the total variance (Kock, 2021).

Data analyses were conducted using SPSS 26.0 and Mplus 8.3. First, descriptive statistics were calculated to summarize participants’ background characteristics and main study variables. Regression analyses were used to examine the associations between background factors and FHSI/IHSI. Pearson correlation analyses were then performed to assess bivariate relationships among social support, self-compassion, resilience, FHSI, and IHSI.

To assess multicollinearity, the Variance Inflation Factor (VIF) was calculated for all predictors. None of the VIF values exceeded the commonly accepted threshold of 10, indicating that multicollinearity was not a concern in the model.

Structural equation modeling (SEM) was employed to test the hypothesized mediation model. Social support was specified as the independent variable, self-compassion and resilience as parallel mediators, and formal and informal help-seeking intentions as outcome variables. Background variables that showed significant associations with FHSI and IHSI were included as covariates in the model. Model fit was evaluated using multiple indices, including the comparative fit index (CFI), Tucker–Lewis index (TLI), root mean square error of approximation (RMSEA), and standardized root mean square residual (SRMR) (Lu et al., 2025a; Ren et al., 2025, 2026). Indirect effects were tested using the bootstrap method with 5,000 resamples, and 95% bias-corrected confidence intervals were generated. An indirect effect was considered statistically significant if the confidence interval did not include zero.

## 3 Results

### 3.1 Results of common method bias test

In this study, the strongest factor extracted from an exploratory factor analysis (EFA) explained 27.4% of the total variance. According to the Harman single-factor criterion, there was no apparent common method bias.

### 3.2 Descriptive analysis

Table 1 presents the results of descriptive analysis. The participants had a mean age of 19.9 years (SD = 1.3). Females accounted for 63.5% of the sample, and 46.2% were juniors. More than half of the students (58.8%) rated their perceived family financial situation as average, and 5.8% reported being from single-parent families. The mean score for social support was 16.9 (SD = 3.1), and the mean for resilience was 5.5 (SD = 1.6). The average self-compassion score was 46.9 (SD = 9.6). The mean score for IHSI was 6.8 (SD = 2.0), whereas the mean for FHSI was 3.3 (SD = 1.3).

**Table 1:** Participants’ characteristics.

Categorical variables	*n*	%
Sex		
Male	250	36.5
Female	434	63.5
Undergraduate levels		
Freshman	168	24.6
Sophomore	116	17.0
Junior	316	46.2
Senior	84	12.3
Perceived family financial situation		
Very poor/poor	120	17.5
Average	402	58.8
Good/very good	162	23.7
Single-parent family status		
No	582	85.1
Yes	40	5.8
Choosing not to report	62	9.1

Continuous variables	Mean	SD
Age	19.9	1.3
Social support	16.9	3.1
Resilience	5.5	1.6
Self-compassion	46.9	9.6
IHSI	6.8	2.0
FHSI	3.3	1.3

IHSI, informal help-seeking intention; FHSI, formal help-seeking intention.

### 3.3 The association between background factors and FHSI/IHSI

Females were also more likely to show higher IHSI than males (*β* = 0.16). Perceived family financial situation was positively associated with IHSI. Compared with students who reported a very poor/poor family financial situation, those who rated their situation as average (*β* = 0.10) or good/very good (*β* = 0.12) showed significantly higher levels of IHSI. In contrast, students from single-parent families were less inclined to seek informal help compared with those from two-parent families (*β* = −0.15).

For FHSI, age was negatively related (*β* = −0.08), indicating that older students were less willing to seek formal help. Females reported higher levels of FHSI than males (*β* = 0.12). Similar to the pattern observed for informal help-seeking, students with average (*β* = 0.13) or good/very good (*β* = 0.15) family financial situations exhibited significantly greater FHSI than those with very poor/poor family financial backgrounds. Such data are presented in Table 2.

**Table 2:** The associations between background factors and IHSI/FHSI.

	IHSI		FHSI	
Variables	*β*	SE	*β*	SE
Age	0.04	0.06	−0.08*	0.04
Sex				
Male	Ref		Ref	
Female	0.16***	0.09	0.12**	0.10
Undergraduate levels				
Freshman	Ref		Ref	
Sophomore	0.01	0.24	0.08	0.15
Junior	0.08	0.19	0.01	0.12
Senior	0.05	0.27	0.02	0.17
Perceived family financial situation				
Very poor/poor	Ref		Ref	
Average	0.10*	0.05	0.13**	0.06
Good/very good	0.12**	0.06	0.15**	0.08
Single-parent family status				
No	Ref		Ref	
Yes	−0.15***	0.03	−0.04	0.21
Choosing not to report	−0.01	0.27	−0.02	0.17

IHSI, informal help-seeking intention; FHSI, formal help-seeking intention; Ref, reference group. *p < 0.05; **p < 0.01; ***p < 0.001.

### 3.4 Pearson correlations

As shown in Table 3, social support, resilience, self-compassion, FHSI, and IHSI were positively correlated with each other, *r* ranges from 0.16–0.56, *ps* < 0.001.

**Table 3:** Pearson correlations.

Variables	1	2	3	4	5
Social support	1				
Resilience	0.37^***^	1			
Self-compassion	0.33^***^	0.56^***^	1		
IHSI	0.51^***^	0.16^***^	0.25^***^	1	
FHSI	0.20^***^	0.20^***^	0.24^***^	0.36^***^	1

IHSI, informal help-seeking intention; FHSI, formal help-seeking intention. *p < 0.05; **p < 0.01; ***p < 0.001.

### 3.5 Results of multicollinearity test

To assess multicollinearity, the Variance Inflation Factor (VIF) was calculated for all predictors in the model. None of the VIF values exceeded the threshold of 10, indicating that multicollinearity was not a concern (Supplementary Table 1).

### 3.6 Mediation results

**Figure 1:**
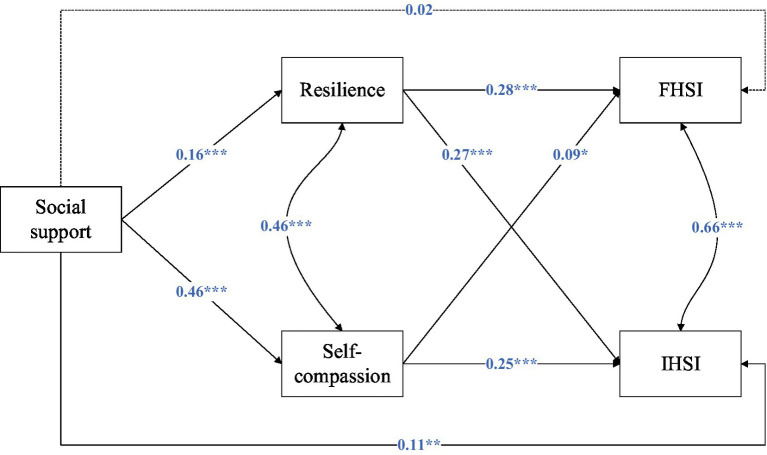
Results of structural equation modeling. The model was adjusted for age, sex, perceived family financial situation and single-parent status. *p < 0.05; **p < 0.01; ***p < 0.001.

Figure 1 and Table 4 present the model testing the mediation effects of self-compassion/resilience between social support and informal/formal help-seeking intention. SEM yielded satisfactory model fit indices [χ^2^(*df*) = 10.30(4), CFI = 0.991, TLI = 0.959, RMSEA = 0.048, SRMR = 0.025].

**Table 4:** Mediation analysis.

Path	*β*	SE	95% CI	*p*
Direct path				
SS → FHSI	0.02	0.04	(−0.06, 0.10)	0.678
SS → IHSI	0.11	0.04	(0.03, 0.19)	0.006
Indirect path				
SS → Resilience → FHSI	0.04	0.01	(0.01, 0.07)	< 0.001
SS → SC → FHSI	0.04	0.01	(0.02, 0.06)	0.038
SS → Resilience → IHSI	0.04	0.01	(0.01, 0.07)	< 0.001
SS → SC → IHSI	0.12	0.02	(0.07, 0.17)	< 0.001

SS, social support; IHSI, informal help-seeking intention; FHSI, formal help-seeking intention; SC, self-compassion.

Social support was not directly associated with FHSI (*β* = 0.02, *p* = 0.678). However, social support showed a positive relationship with resilience (*β* = 0.16, *p* < 0.001), which, in turn, was positively related to FHSI (*β* = 0.28, *p* < 0.001). The indirect effect through resilience was statistically significant (*β* = 0.04, *p* < 0.001), suggesting that resilience mediated the link between social support and FHSI. Similarly, social support was positively associated with self-compassion (*β* = 0.46, *p* < 0.001), and self-compassion was positively related to FHSI (*β* = 0.09, *p* = 0.023). The indirect effect via self-compassion was also significant (*β* = 0.04, *p* = 0.038). Notably, the path coefficient from social support to self-compassion was substantially larger than that to resilience, indicating that perceived social support may be more readily translated into self-acceptance-related capacities than into general psychological resilience. Together, these results indicate that resilience and self-compassion fully mediated the relationship between social support and FHSI.

In contrast, social support was directly and positively associated with IHSI (*β* = 0.11, *p* = 0.006). Social support was again positively related to resilience (*β* = 0.16, *p* < 0.001), which, in turn, was associated with higher IHSI (*β* = 0.27, *p* < 0.001). The indirect effect through resilience was significant (*β* = 0.04, *p* < 0.001). In addition, social support was positively related to self-compassion (*β* = 0.46, *p* < 0.001), and self-compassion was strongly associated with IHSI (*β* = 0.25, *p* < 0.001). The mediation analysis confirmed a significant indirect effect through self-compassion (*β* = 0.12, *p* < 0.001). These findings suggest that resilience and self-compassion partially mediated the association between social support and IHSI.

## 4 Discussion

This study investigated how social support relates to both FHSI and IHSI regarding MPA among Chinese music students, focusing on the mediating roles of resilience and self-compassion. Results showed that social support was not directly linked to FHSI; instead, higher social support was associated with higher resilience and self-compassion, which were in turn positively associated with FHSI. The indirect paths through resilience and self-compassion were statistically significant, supporting full mediations. For IHSI, social support displayed a positive direct association and, simultaneously, positive indirect associations via resilience and via self-compassion; both indirect paths were significant, indicating partial mediation. These patterns highlight the joint contribution of interpersonal resources and personal capacities to music students’ help-seeking intentions regarding MPA.

Sex differences emerged consistently across help-seeking types, with female students reporting higher FHSI and IHSI than male students. This pattern aligns with previous research indicating that women tend to express emotions more openly and experience less stigma when seeking interpersonal or professional support (Qiu et al., 2024). In contrast, male students may be more influenced by self-reliance norms, which can discourage help-seeking behaviors. These findings suggest the need for gender-sensitive interventions that normalize and encourage help-seeking among male students (McDermott et al., 2018). Perceived family financial situation and family structure also showed significant associations with FHSI and IHSI. Students who rated their family financial situation as average or good/very good were more likely to seek both formal and informal help than those reporting poor or very poor conditions. A positive financial perception may enhance individuals’ sense of efficacy, perceived access to services, and tolerance for potential costs of help-seeking (Rowan et al., 2013). Meanwhile, students from single-parent families reported lower IHSI, possibly due to reduced emotional resources, limited caregiver time, or stronger internalized independence norms (Lu et al., 2025b). Institutions could help mitigate these challenges by offering low-threshold, confidential, and peer-based mental health services tailored for students from disadvantaged backgrounds.

The mediating role of self-compassion in the relationship between social support and IHSI (H1) suggests that perceived social support enhances students’ capacity for self-kindness and acceptance, thereby reducing self-criticism and emotional suppression. Within the conservatory environment, where self-judgment and fear of evaluation are pervasive, self-compassion allows students to reinterpret emotional vulnerability as a normal and shared human experience rather than a personal failure (Neff, 2023). When individuals perceive warmth and acceptance from others, they internalize this acceptance and extend it toward themselves, which lowers anticipated shame and facilitates emotional disclosure to peers or close others (Sajadian et al., 2024). Particularly in the Chinese sociocultural context, where maintaining face and avoiding burdening others are culturally salient values, self-compassion may serve as an intrapersonal buffer that legitimizes emotional expression, thereby transforming social support into a psychological permission structure for informal help-seeking.

Similarly, the significant mediation of resilience in the association between social support and IHSI (H2) reveals that social connections foster adaptive coping capacities that encourage constructive engagement rather than avoidance. When students feel supported, they develop greater confidence in their ability to recover from stressors, viewing help-seeking as an active and resourceful response rather than a signal of inadequacy (Cao et al., 2024). Resilience reframes the experience of MPA from an uncontrollable threat to a manageable challenge, promoting approach-oriented coping behaviors such as seeking advice, feedback, or emotional validation from peers (Zhang and Jenatabadi, 2024). This interpretation is consistent with recent university-level research showing that performance-related anxiety can function as an intermediate mechanism linking psychological resources to adaptive academic outcomes, thereby supporting the logic of indirect pathways from internal resources to functional responses (Vasiou et al., 2025). Within the context of intensive musical training, resilience allows students to interpret seeking help as part of performance optimization (Arbinaga, 2023). It becomes a pragmatic strategy to enhance self-regulation and maintain artistic excellence, aligning help-seeking with the values of mastery and professionalism that characterize performance cultures (Mazzarolo et al., 2023).

The mediation of self-compassion in the relationship between social support and FHSI (H3) further emphasizes the role of self-directed kindness in overcoming internalized stigma. Formal help-seeking typically involves greater perceived risks, such as labeling, fear of being seen as weak, or concerns about professional reputation, especially in collectivistic cultures that prize self-control and perseverance (Ntumi et al., 2025). Social support signals social acceptance and normalizes emotional vulnerability, while self-compassion transforms this external validation into internal permission to seek professional care without self-blame (Chan and Tsui, 2025). By reducing shame and self-stigma, self-compassion allows students to view professional help not as an admission of failure but as a responsible act of self-care and growth (Cepni et al., 2024). This is particularly crucial for music students in China, where concerns about reputation and teacher evaluations often deter engagement with psychological services. Self-compassion thus acts as a moral and emotional bridge, converting the normative approval embedded in social support into genuine openness to formal mental health resources.

The mediating effect of resilience on the relationship between social support and FHSI (H4) reveals a complementary yet distinct psychological process. Whereas self-compassion primarily mitigates affective barriers to help-seeking, resilience fosters cognitive and behavioral readiness to take purposeful action. Resilient students are more likely to construe formal help as a strategic and competent response to stress, aligning with a problem-solving orientation rather than a defeatist narrative (Litt et al., 2025). Through resilience, the benefits of social support are internalized as a belief in one’s capacity to manage challenges effectively, which enhances motivation to engage with professional interventions when self-regulation alone is insufficient (Lu et al., 2025b). Within performance-oriented academic settings, resilient students tend to perceive counseling or psychological services as opportunities to refine regulation skills and maintain artistic functioning. This adaptive interpretation aligns help-seeking with the values of discipline and mastery, thereby integrating professional support into the broader framework of musical and personal development.

Taken together, the contrast between full mediation for FHSI and partial mediation for IHSI reflects a theoretically and culturally meaningful distinction. From the perspective of Stress and Coping Theory (Lazarus and Folkman, 1984), formal help-seeking represents a higher-cost coping option that requires more extensive secondary appraisal, including evaluations of self-worth, stigma, and coping competence. In the Chinese cultural context, strong face concerns and norms of emotional restraint may prevent social support from directly translating into formal help-seeking. Instead, social support appears to exert its influence by first enhancing self-compassion to reduce self-criticism and by strengthening resilience to increase perceived coping capacity, thereby indirectly facilitating engagement with professional help. In contrast, informal help-seeking from friends and family involves lower perceived social risk and aligns more closely with relational norms, allowing social support to exert both direct and indirect effects. This distinction helps explain why social support alone is sufficient to encourage informal help-seeking, whereas formal help-seeking depends more heavily on the transformation of external support into internal psychological resources.

Beyond the distinction between formal and informal help-seeking, our findings further indicate that self-compassion and resilience play different but related roles in how social support is associated with help-seeking intentions among Chinese music students. Self-compassion seems to work mainly on an emotional level, easing feelings of shame and fear that often prevent students from talking about their anxiety or seeking help. Resilience, on the other hand, strengthens a sense of control and the belief that challenges can be managed through active coping. In this way, social support is not only a source of comfort but also a foundation for developing internal motivation to seek help when needed. It is also possible that additional untested factors, such as perceived stigma, performance-related perfectionism, or coping style, may play parallel or sequential mediating roles (Xiong et al., 2024; Lu et al., 2025b). Future research could explore whether these variables interact with self-compassion and resilience to shape help-seeking processes more comprehensively. It should also be noted that, due to the cross-sectional design, alternative temporal sequences among the variables cannot be ruled out. For example, self-compassion or resilience may precede and shape individuals’ perceptions of available social support, or these personal and interpersonal resources may develop in parallel over time. Within the conservatory environment, where emotional restraint and achievement orientation are deeply ingrained, fostering such internal resources may help students reinterpret help-seeking as both culturally appropriate and instrumental to artistic growth.

While the statistical significance of the paths between social support, resilience, self-compassion, and help-seeking intentions was established, the relatively small effect sizes should be interpreted cautiously. This implies that, while the results are statistically significant, the practical impact of these findings in real-world applications may be limited, especially considering the small-to-moderate effect sizes. Therefore, interventions should not only focus on statistical outcomes but also consider the real-world relevance of these effects, ensuring that interventions lead to meaningful, actionable change.

From a practical standpoint, these findings suggest that interventions to alleviate MPA should address both relational and psychological dimensions. Programs that strengthen peer and teacher support systems may create the interpersonal foundation necessary for self-compassion and resilience to develop (Hu, 2023). Simultaneously, integrating self-compassion and resilience training into performance pedagogy through workshops, reflective practice, or mindfulness-based modules could enhance students’ capacity to engage with both informal and formal help-seeking pathways (Czajkowski et al., 2022). Ultimately, by illuminating the internal mechanisms linking social support to adaptive help-seeking, this study contributes to a more holistic understanding of mental health promotion in performance education and offers a culturally sensitive framework for supporting Chinese music students’ emotional well-being.

Despite its strengths, this study has several limitations. First, all variables were measured through self-report questionnaires, which may be affected by social desirability bias and response subjectivity. In particular, students may underreport psychological distress or overreport adaptive coping tendencies due to concerns about social evaluation. Second, the cross-sectional design limits causal interpretation, and the observed associations cannot be interpreted as evidence of directional or causal relationships; longitudinal or intervention studies are therefore needed to confirm the proposed relationships and examine changes in help-seeking intentions over time. Third, prior help-seeking behaviors and other potential mediators, such as perceived stigma, perfectionism, or coping style, were not included and may have influenced the observed associations. Fourth, the study examined help-seeking intentions rather than actual behaviors, and there is often a discrepancy between intention and action. In addition, although the brief resilience scale and the single-item measure of FHSI used in this study have demonstrated acceptable psychometric properties and are commonly adopted in large-scale survey research, their brevity may limit the conceptual breadth with which these constructs are captured. Finally, the sample consisted of music students from a limited number of Chinese conservatories, which may restrict the generalizability of the findings. Future research should address these limitations by employing longitudinal or multi-source designs and by extending the proposed model to students in other artistic domains (e.g., theatre, dance, or visual arts), or through comparative studies across different forms of arts education, to examine whether the identified mechanisms operate similarly across artistic contexts.

## 5 Conclusion

This study examined how social support influences formal and informal help-seeking intentions for MPA among Chinese music students, emphasizing the mediating roles of resilience and self-compassion. The results demonstrated that social support indirectly promoted formal help-seeking through both mediators, while its effect on informal help-seeking was partly direct and partly mediated. These findings suggest that the benefits of social support extend beyond external reassurance, functioning through the development of inner psychological resources that motivate proactive coping and help-seeking behavior. By identifying resilience and self-compassion as key mechanisms, this study contributes to a deeper understanding of how interpersonal and intrapersonal factors jointly shape students’ responses to performance-related distress. Practically, the results support the value of integrating social, emotional, and psychological skill development into conservatory training to foster adaptive coping and encourage help-seeking as a normal component of professional musicianship.

## Data Availability

The raw data supporting the conclusions of this article will be made available by the authors, without undue reservation.
