# Supplementary material for: Associations between social support and help-seeking intention regarding music performance anxiety among Chinese music students: mediation via resilience and self-compassion
Source: Front Psychol. 2026 Feb 12;17:1787708. doi: 10.3389/fpsyg.2026.1787708 (PMC12935999; doi:10.3389/fpsyg.2026.1787708)
Supplement: Supplementary file 1 [file Table_1.DOCX]

Supplementary Table 1 VIF and tolerance for Study Variables

| Variable | IHSI | | | FHSI | |
| --- | --- | --- | --- | --- | --- |
|  | VIF | Tolerance | VIF | | Tolerance |
| Social support | 1.19 | 0.84 | 1.19 | | 0.84 |
| Resilience | 1.53 | 0.65 | 1.53 | | 0.65 |
| Self-compassion | 1.49 | 0.67 | 1.49 | | 0.67 |

VIF = Variance Inflation Factor, IHSI = Informal help-seeking intention, FHSI = formal help-seeking intention.
